# Scaling up = scaling down? Children’s spatial scaling in different perceptual modalities and scaling directions

**DOI:** 10.1186/s41235-023-00517-7

**Published:** 2023-10-04

**Authors:** Wenke Möhring, Magdalena Szubielska

**Affiliations:** 1 Department of Psychology, University of Basel, Missionsstrasse 62, 4055 Basel, Switzerland; 2 Department of Educational and Health Psychology, University of Education Schwäbisch Gmünd, Schwäbisch Gmünd, Germany; 3 Institute of Psychology, Faculty of Social Sciences, The John Paul II Catholic University of Lublin, Lublin, Poland

**Keywords:** Spatial scaling skills, Visual modality, Haptic modality, Scaling direction, Children, Development

## Abstract

The present study examined whether scaling direction and perceptual modality affect children’s spatial scaling. Children aged 6–8 years (*N* = 201) were assigned to a visual, visuo-haptic, and haptic condition in which they were presented with colourful, embossed graphics. In the haptic condition, they were asked to wear a blindfold during the test trials. Across several trials, children were asked to learn about the position of a target in a map and to localize a disc at the same location in a referent space. Scaling factor was manipulated systematically, so that children had to either scale up or scale down spatial information. Their absolute deviations from the correct target location, reversal and signed errors, and response times served as dependent variables. Results revealed higher absolute deviations and response times for the haptic modality as opposed to the visual modality. Children’s signed errors, however, showed similar response strategies across the perceptual conditions. Therefore, it seems that a functional equivalence between vision and touch seems to emerge slowly across development for spatial scaling. With respect to scaling directions, findings showed that absolute deviations were affected by scaling factors, with symmetric increases in scaling up and scaling down in the haptic condition. Conversely, children showed an unbalanced pattern in the visual conditions, with higher accuracy in scaling down as opposed to scaling up. Overall, our findings suggest that visibility seems to factor into children’s scaling process.

## Introduction

Spatial scaling allows relating spatial information in different-sized spaces (Frick & Newcombe, 2012) and is required when connecting information between a small- (or large-) scaled model and the real-life referent it stands for. This ability is involved in several tasks in daily life (e.g. when constructing new furniture in accordance with a small-scaled assembly instruction), in educational institutions (e.g. when relating a large-scaled model of an insect to the real-life referent), or in professions (e.g. when interpreting a small-scaled blueprint for a building on a construction site). Several studies have shown that spatial scaling is related to children’s mathematical abilities (e.g. Boyer & Levine, 2012; Frick, 2019; Jirout et al., 2018; Mix et al., 2016; Möhring et al., 2018) and their science achievement (Hodgkiss et al., 2018), highlighting the importance of this particular spatial skill for children’s academic achievement. Moreover, a recent training study suggested causal effects of spatial scaling on children’s mathematical achievement, as a short training of children’s spatial scaling improved their number line estimations (Gilligan et al., 2020).

### Scaling up versus scaling down

As can be seen in the examples mentioned above, spatial scaling involves different scaling directions. For example, we scale spatial information up from a small-scaled model to the referent it stands for (e.g. when scaling up information provided in a map to a city). Likewise, we scale spatial information down from a large-scaled model to the referent it stands for (e.g. when scaling down information provided in a large-scaled model in a science museum to the real-life referent). Children are constantly confronted with these scaling directions in their daily life. However, until now, the majority of scaling studies has focused on investigating scaling up (e.g. Frick & Newcombe, 2012; Huttenlocher et al., 1999; Möhring et al., 2014, 2018; Vasilyeva & Huttenlocher, 2004). Given the frequent usage and high relevance, investigations involving both scaling directions are timely and would enable conclusions about the flexibility of the scaling process.

An early study investigating this topic suggested differences between these scaling directions (e.g. Siegel et al., 1979). In this study from Siegel et al. (1979), children were first presented with a small-scaled model of a town and then asked to reproduce the layout of this town in a larger room, investigating children’s ability to scale up spatial information (using a scaling factor of 1:6). In a similar scaling down condition, children were first presented with a model of the town in a large room and then asked to reproduce the same layout in a small-scaled referent space (using a scaling factor of 1:6). The authors found that children performed more accurately in scaling down as compared to scaling up and concluded that different processes might be involved for different scaling directions.

As can be seen in this respective study, a crucial precondition when investigating effects of scaling direction concerns the importance of keeping the size of the referent space constant across different scaling directions. Naturally, larger rooms are associated with more room for errors as compared to smaller referent spaces (for a discussion, see Vasilyeva & Huttenlocher, 2004). Thus, the results from Siegel et al. (1979) might be explained by this larger referent space in case of scaling up as opposed to scaling down. Recent studies which addressed this methodological requirement revealed similar results for each scaling direction (in a discrimination paradigm with adults: Möhring et al., 2016; in a localization task with children: Plumert et al., 2019, Exp. 3), indicating comparable underlying processes for scaling up and scaling down.

Another influential factor when scaling up and scaling down concerns the absolute size of the referent space. In more recent studies (Hund et al., 2020; Plumert et al., 2019), children and adults were presented with different scaling directions and were asked to learn about the location of an object presented on a learning mat and then to reproduce the same location on a test mat. Across several experiments and scaling directions, it was found that the process of a scale translation between the two mats was impaired when the absolute size of the referent space was very large and the edges of the test mat were hard to see at a single moment (called the perceptual anchoring effect). Following the authors’ explanation, participants may have generated a mental representation of the learning mat which was then compared to the test mat. The test mat thus acted as a perceptual anchor. If this anchor was very large, participants needed to make head or eye movement to encode the entire space, which may have disrupted the scale translation and result in higher errors.

Consequently, it seems that several factors influence scaling performance along different directions and thus should be carefully considered when investigating effects of scaling directions. Based on these previous studies (Hund et al., 2020; Plumert et al., 2019; Siegel et al., 1979), the referent space should be same-sized for both scaling directions which has been addressed rather seldomly in previous research (for an exception, see Plumert et al., 2019). Furthermore, the edges of the referent space should be easily viewable without the need for eye or head movements to avoid interference effects on the scaling process (cf. Hund et al., 2020; Plumert et al., 2019). Finally, scaling direction (up vs. down) should be preferably manipulated in a within-participant design, with children being tested with both directions, allowing to directly compare their performance when scaling up and scaling down. None of the previous studies has used such a within-participant design (Hund et al., 2020; Plumert et al., 2019; Siegel et al., 1979). Thus, we decided to implement this methodological approach and to address the other points in the present study.

### Scaling in different perceptual modalities

Another question that has been hardly investigated up to now concerns scaling in different perceptual modalities. So far, research investigating children’s spatial scaling has predominantly focused on the visual domain (Frick & Newcombe, 2012; Huttenlocher et al., 1999; Möhring et al., 2014, 2015, 2018; Plumert et al., 2019; Vasilyeva & Huttenlocher, 2004). These studies have typically used localization tasks, in which children were presented with map-like spaces showing a target and were asked to visually encode this map (similar to the learning mat in Hund et al., 2020; Plumert et al., 2019). Afterwards, they were asked to reproduce the same location in a different-sized referent space. Maps and referent spaces were systematically varied in size (i.e. using multiple scaling factors), so that participants needed to scale spatial information. Several studies showed that scaling factors affected children’s absolute errors and response times (RTs), such that larger scaling factors were associated with higher errors and RTs (e.g. Möhring et al., 2014; Plumert et al., 2019; Vasilyeva & Huttenlocher, 2004). More concrete, it was even shown that errors and RTs varied linearly with higher scaling factors. In analogy to mental imagery research (e.g. Kosslyn, 1975; Shepard & Metzler, 1971), this result pattern has been interpreted as indicating mental transformation strategies in spatial scaling. Participants may mentally shrink or enlarge their mental representations of the map (i.e. they zoom in or out this mental image) and transfer this information to the referent space (cf. Möhring et al., 2014).

Importantly, even though scaling has been predominantly investigated in the visual domain, it is not restricted to this modality. Another modality that also allows encoding spatial information concerns the haptic sense. In analogy to the visual sense, it is equally possible to receive a variety of spatial information about an object when exploring it by touch (e.g. size, orientation, shape, texture, relations to other objects). But naturally, exploring objects haptically requires more time as participants have to serially explore the object’s characteristics as opposed to a quicker exploration in the visual modality. Furthermore, haptic exploration is limited to the observer’s peripersonal space (i.e. the space around the body).

Despite these differences between perceptual modalities, spatial information can be similarly scaled along the visual and haptic sense. The crucial question is whether mental imagery is also used in haptic scaling. Investigating this question is important as findings would support theories proposing a functional equivalence of spatial representations from vision and touch (Giudice et al., 2011; Loomis et al., 2013). These studies suggest amodal spatial representations instead of modality-specific representations. Thus, investigating this question allows understanding the nature of human’s mental representations. Indeed, several adult studies investigating spatial scaling have yielded evidence in favour of this functional equivalence of vision and touch considering that adults’ RTs and errors varied systematically as a function of scaling factors (Szubielska & Möhring, 2022; Szubielska et al., 2022a). However, the developmental perspective onto this functional equivalence remains widely unexplored.

To our knowledge, there is only a single study that investigated *children’s* spatial scaling in the haptic domain so far (Szubielska et al., 2019). This respective study comprised samples of congenital blind and sighted blindfolded participants in an age range from 8.5 to 45 years. It was found that participants’ absolute errors increased with larger scaling factors in congenital blind participants but not in sighted, blindfolded participants. Thus, it seems that at least in samples with much experience in haptic exploration (i.e. blind participants), performance resembled previous findings in the visual domain (e.g. Möhring et al., 2014). However, the sample comprised mainly adolescents and adults (74%, *n* = 34), with only few children being involved (*n* = 12). Furthermore, no separate analyses for children and adults were available. Therefore, until now, it seems unclear whether children’s spatial scaling in the haptic domain resembles their scaling in the visual domain.

Other studies have not particularly focused on spatial scaling but investigated related capabilities such as recognizing maps and objects in the visual and haptic domain (Craddock & Lawson, 2009a, b; Giudice et al., 2011; Srinivas et al., 1997). All of these studies have been conducted with adult samples. Several studies suggested remarkable similarities in adults’ performance in the visual and haptic modalities (Giudice et al., 2011; Loomis et al., 2013), with high overlap in participants’ responses across the two domains. Other studies have revealed that haptic exploration seems more error-prone as compared to the visual modality (e.g. in the complex condition in Ottink et al., 2021), with participants producing higher absolute errors and RTs in the haptic condition. In sharp contrast, there is also one study indicating that estimations about spatial areas were more accurately in the haptic domain as compared to the visual domain (e.g. Intraub et al., 2015). Therefore, previous findings seem rather heterogeneous for adults. Importantly, however, children’s spatial scaling in the haptic domain remains understudied as of today.

### The present study

Goals of the present study were twofold. As a first aim, we sought to understand effects of scaling direction on children’s absolute deviations and RTs as both scaling directions are used frequently in daily life. Yet, the flexibility of these processes remains poorly understood in children (Hund et al., 2020; Plumert et al., 2019; Siegel et al., 1979). Building upon these previous studies, we manipulated scaling direction in a within-participant design and took care to keep the size of the referent space same-sized and easily viewable across the conditions. Following findings of previous research (e.g. Plumert et al., 2019; Exp. 3), we expected similar (symmetric) effects on scaling performance irrespective for scaling up and scaling down.

As a second aim, we sought to compare children’s spatial scaling between the visual and haptic modality and were interested in similarities and differences when being confronted with these perceptual conditions. We added another bimodal condition, in which visual and haptic exploration was combined, to see whether receiving information via both senses would be beneficial or detrimental for children’s scaling. Children were randomly assigned to these three perceptual conditions. In each condition, a recently developed methodology was used to assess spatial scaling (cf. Szubielska et al., 2022a). First, children were presented with a map showing a target and asked to memorize the target’s location. Immediately afterwards, they were presented with a referent space and asked to reproduce the same location on a referent space by locating a disc. Sizes of maps and referent spaces differed systematically, and thus, different scaling factors were used. Using this two-stage procedure, we measured scaling from memory (for similar approaches, see Intraub et al., 2015; Plumert et al., 2019). This approach was identical across the three perceptual conditions and allowed us to reliably assess participants’ absolute deviations and RTs, irrespective of the expected outcome that participants may need longer to explore spatial layouts in the haptic condition (for a discussion, see Szubielska & Möhring, 2019). Furthermore, we used colourful and embossed graphics, enabling to easily explore these graphics by vision and touch.

In the haptic condition, children were blindfolded for the time of each trial. There are several studies showing that blindfolds have been used successfully in young children (5- to 16-year-olds in Bedny et al., 2015; 4- to 8-year-olds in Morrongiello et al., 1995; 6- to 14-year-olds in Vinter et al., 2018). Nevertheless, we were aware that children might feel uncomfortable with being blindfolded. For this reason, we aimed at testing slightly older children aged 6–8 years as compared to previous research on spatial scaling (e.g. 4-year-olds in Möhring et al., 2014; Plumert et al., 2019). This particular age range was chosen given that spatial scaling skills seem to develop considerably up to the age of 8 years (e.g. Gilligan et al., 2018).

Building upon theories proposing function equivalence between vision and touch (Giudice et al., 2011; Loomis et al., 2013), we expected similar outcomes for the visual, visuo-haptic, and haptic condition. That is, RTs and absolute deviations may vary as a linear function of different scaling factors, with higher response times and larger absolute deviations for larger scaling factors. However, given the predominance of the visual modality (e.g. Hutmacher, 2019; Ottink et al., 2021), we expected higher absolute deviations and RTs in the haptic condition as opposed to both visual conditions. Furthermore, we were interested in children’s response strategies in each perceptual domain. Exploring children’s biases towards reference points across different perceptual modalities may reveal further evidence for the functional equivalence theory. Based on previous research in the visual domain (Huttenlocher et al., 1991, 1994), we expected that children might separate the space into two halves and gravitate towards the midpoint in each half.

## Methods

### Participants

A total of 201 6- to 8-year olds participated in the present study (for demographic and descriptive variables, see Table 1). Two additional children were tested but had to be excluded because they did not comply with the task instructions. Children attended Swiss and German kindergartens and schools. The majority of children was White. Participants were excluded from study participation when they were diagnosed with developmental disorders such as autism-spectrum disorder and when the children’s level of German language skills was not fluent enough to ensure that they understood the task instructions. The ethics committee of the respective University approved the current study (protocol number: 004-21-1). Parents answered demographic questions and questions related to handedness by filling in an online questionnaire. Children provided verbal assent prior to study participation and parents gave written informed consent. Children and their families received a voucher for their participation.

**Table 1:** Demographic details of the present sample and descriptive statistics of the WISC-V standardized subtests

	6 years (*n* = 59)	7 years (*n* = 82)	8 years (*n* = 60)
	*N* (%)/*M* (*SD*)	*N* (%)/*M* (*SD*)	*N* (%)/*M* (*SD*)
Age in years	6.57 (0.28)	7.53 (0.27)	8.33 (0.28)
Female	32 (54.2%)	37 (45.1%)	20 (33.3%)
Right-handed^a^	48 (81.4%)	75 (91.5%)	49 (81.7%)
Maternal education^b^			
No school degree	0 (0%)	1 (1.3%)	0 (0%)
Mandatory school	1 (1.8%)	2 (2.5%)	2 (3.6%)
Apprenticeship	5 (9.1%)	9 (11.4%)	3 (5.4%)
High school	1 (1.8%)	5 (6.3%)	3 (5.4%)
Higher education	3 (5.5%)	6 (7.6%)	6 (10.7%)
University /college	45 (81.8%)	56 (70.9%)	42 (75%)
WISC-V vocabulary^c^	11.76 (3.41)	12.04 (3.09)	11.62 (3.04)
WISC-V matrices^c^	11.02 (2.43)	10.54 (2.44)	11.43 (2.61)

^a^No data were available for *n* = 10 children

^b^No data were available for *n* = 11 mothers

^c^Refer to age-standardized scores with *M* = 10, *SD* = 3

A priori power analyses with G-Power 3 (Faul et al., 2007) were based on previous studies with adults (Intraub et al., 2015; Szubielska & Möhring, 2019; Szubielska et al., 2022a), given that research investigating children’s spatial scaling in different perceptual modalities is rare. Assuming a comparable, moderate-to-strong effect size of *f* = 0.32, significance levels of *p* < 0.05, and a power of 0.80, revealed a minimum sample size of 60 children per age group (amounting to a total of 180 children). Thus, our sample seems adequately powered to investigate the present research questions.

### Measures

Children were tested individually within a single experimental session by trained research assistants. They were tested in a quiet, separate room at their educational institution or in a laboratory at the respective University. To assess children’s typical cognitive development and the representativity of the present sample, children were first presented with two subtests from the Wechsler Intelligence Scale for Children-5th Edition (Wechsler, 2017). Another reason for conducting these subtests at the beginning was to familiarize children with the experimental situation and to establish a good relationship between the experimenter and the child. This seemed important given that the haptic condition of the scaling task involved wearing a blindfold. The WISC-V is a well-acknowledged instrument assessing several components of intellectual ability and complies high reliability and validity standards (for a critical review, see Canivez & Watkins, 2016). Children were examined with the subtests “vocabulary” and “matrix reasoning”. Performances on these subtests are seen as proxies for children’s verbal reasoning and fluid intelligence, respectively (Groth-Marnat, 2009). As can be seen in Table 1, on average, results indicate typical development in each age group.

Furthermore, children took part in a spatial scaling task which was embedded in a story about a dog who likes hiding his toys in the garden. In this task, children were asked to help the dog with finding the toys. Participants were presented with a map showing where the toy was located and a referent space in which children were asked to indicate where the toy was located using a small disc. In the same experimental session, children also took part in other tasks that are beyond the scope of the present study and are described elsewhere (e.g., Möhring et al., 2022).

#### Material in the spatial scaling task

**Fig. 1:**
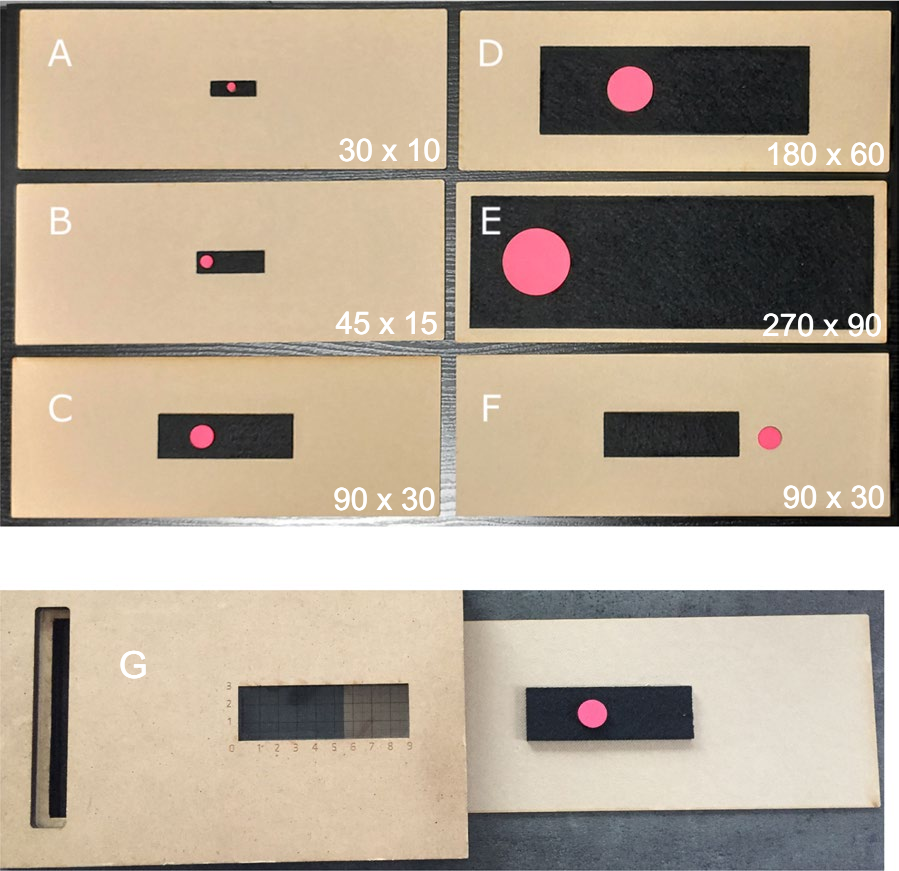
Photographs of the materials used in the current study. **A**–**E** refer to the maps in the five scaling factor conditions. **F** refers to the referent space with the disc. Dimensions of the spaces are included for each map and referent space in mm. **G** refers to the apparatus with an integrated coordinate system used for coding the coordinates of participant’s answers (the referent space could be inserted into the apparatus and was fixed while the experimenter took a picture)

The map was a rectangular space made of black felt which was glued upon a wooden board. Fixed upon on this black felt, there was a target which was represented by a round, convex circle made of pink sponge rubber. This target was presented in one of five equidistant locations on this rectangular space (see Table 2, for *x*- and *y* coordinates; see Fig. 1 for pictures). Material and colors of the target and space were very distinct and thus could be easily explored by touch and sight. Importantly, such a collage technique in embossed graphics has been shown to increase accuracy of haptic encoding as opposed to a raised-line technique (Theurel et al., 2013). Target locations on the maps were centred on the y-axis of the map and thus varied on the horizontal dimension only. As can be seen in Fig. 1, maps had five different sizes which accorded to the scaling factors 1:3, 1:2, 1:1, 2:1, 3:1 (ranging from 30 × 10 mm to 270 × 90 mm).

**Table 2:** Stimuli sizes and coordinates of the targets presented in the maps

Scaling factor	Target diameter (mm)	Map sizes (dimensions of the black rectangle) (mm)		Coordinates of the target position (mm)		Target location	Map number	Pre-determined fixed order
		*X*	*Y*	*X*	*Y*			
1:3	5	30	10	5	5	EL	1	15
				10	5	L	2	21
				15	5	M	3	10
				20	5	R	4	2
				25	5	ER	5	19
1:2	7.5	45	15	7.5	7.5	EL	6	22
				15	7.5	L	7	16
				22.5	7.5	M	8	6
				30	7.5	R	9	11
				37.5	7.5	ER	10	20
1:1	15	90	30	15	15	EL	11	23
				30	15	L	12	17
				45	15	M	13	7
				60	15	R	14	8
				75	15	ER	15	13
2:1	30	180	60	30	30	EL	16	3
				60	30	L	17	5
				90	30	M	18	1
				120	30	R	19	12
				150	30	ER	20	9
3:1	45	270	90	45	45	EL	21	14
				90	45	L	22	4
				135	45	M	23	25
				180	45	R	24	18
				225	45	ER	25	24

The referent space was similarly constructed as the map with the exception that the referent space included no target. Thus, the convex space was made of black felt, which was again glued upon a wooden board. The referent space was same-sized across each trial (90 × 30 mm). Sizes of the map and referent space were the same in scaling factor 1:1; sizes of the other maps increased or decreased systematically, allowing to systematically investigate effects of scaling up (1:2, 1:3) versus scaling down (2:1, 3:1) within a single design. Considering that previous research indicated that scaling is more difficult when participants cannot view both edges of the spaces at once (Hund et al., 2020; Plumert et al., 2019), we took care to include sizes that can be encoded simultaneously. The referent space was always presented together with the disc (diameter of 15 mm), located to the right of the black felt at the beginning of each trial (see Fig. 1, picture F). Participants were instructed to use this disc to indicate the target’s location.

#### Design

Scaling factors (5) and target locations (5) were combined using a full-factorial design, amounting to 25 rectangular maps (for similar approaches, cf. Huttenlocher et al., 1999; Möhring et al., 2014; Vasilyeva & Huttenlocher, 2004). Participants in each age group were randomly assigned to the three perceptual conditions: visual (*n* = 70), haptic (*n* = 65), and visuo-haptic (*n* = 66) condition. In the haptic condition, children were asked to wear a blindfold for the time of each trial. In the visual conditions, children were allowed to visually explore the maps and additionally invited to explore the map by touch in the visuo-haptic condition.

#### Procedure

In each perceptual condition, the spatial scaling task began with three practice trials, followed by 25 test trials that were presented in a predetermined randomized order (see Table 2). Locations in these practice trials differed from the ones in subsequent test trials. Each trial followed a two-stage structure: (1) learning about the map without a time limit, and (2) locating the disc on the empty referent space in accordance with the information provided in the map (cf. Intraub et al., 2015; Plumert et al., 2019, for similar step-wise procedures). At the stage of learning about the map, children were presented with the map and instructed to encode the target location and to remember this location. They were asked to work as quickly and accurately as possible. In the visual and the visuo-haptic perceptual condition, the experimenter placed the covered map in front of the participant. The map was then uncovered and the experimenter started measuring the time from this moment until participants signalled that they had encoded the location by saying “ready”, whereupon the map was taken away. In the haptic perceptual condition, the experimenter measured the time from the moment participants began to touch the map until participants signalled that they had learned the location whereupon the map was taken away. The experimenter measured the learning RTs by pressing a computer key, using a programme written in Cedrus Superlab 4.5.

Immediately afterwards, participants were presented with the constant-sized referent space and asked to locate the disc at the same location as the target presented in the map. Given that maps differed in size, participants had to scale their memorized spatial information and transfer this information to the referent space. The experimenter measured the scaling RTs of this second stage similarly to the learning stage. This phase included the scaling process but also involved localizing the disc on the referent space. Given that this movement of localizing the disc was identical across all trials, we assumed that variation in scaling RTs would be reflective of the scaling process instead of the motor response of localizing the disc. After children had located the disc, the experimenter inserted this referent space including the disc into an apparatus with an integrated coordinate system (see Fig. 1, picture G). The referent space was fixed below this coordinate system and the experimenter took a picture of each child’s response. Later, another research assistant noted the *x*- and *y*-coordinates of each response focusing on the disc’s midpoint in mm. These coordinates were again controlled by another independent research assistant (following the four-eyes principle) and corrected if necessary. To assess inter-rater reliability, we re-coded approx. 25% of children’s responses of the entire sample (*n* = 54) which showed high overlap with the original data (ICC = 0.989, one-way random effects model, single rater). Based on these *x*- and *y*-coordinates, we computed the Euclidean distance between children’s responses and the correct target location (i.e. absolute deviation in mm).

Overall, there were three dependent variables in the spatial scaling task: learning RTs, scaling RTs, and absolute deviations. In addition to absolute deviation, the spatial scaling task offers possibilities to investigate children’s types of errors more closely. In analogy to previous studies (e.g. Frick & Newcombe, 2012; Möhring et al., 2014), we focused on children’s *reversal errors* as an index of children’s propensity to mix up the left and right side of the spatial layout. We considered children’s responses given on the right side of the board (i.e. *x*-coordinate of the response > 45 mm) as reversal errors for targets originally presented on the left side of the map (as seen from the midpoint). Likewise, responses given on the left side of the board (i.e. *x*-coordinate of the response < 45 mm) were coded as reversal errors for targets originally presented on the right side of the map. Furthermore, several studies reported children’s *signed errors* (e.g. Frick & Newcombe, 2012; Plumert et al., 2019), which indicate children’s response biases when locating the targets. These signed errors can help us understanding whether children tend to gravitate their responses to (imagined) reference points such as the midpoint of the space or borders. Signed errors were computed as differences between the *x*-coordinate of the correct (true) target location from the *x*-coordinate of the participant’s responses (in mm, for similar procedures, cf. Frick & Newcombe, 2012; Szubielska & Möhring, 2019). Positive values indicate that the response disc was placed too far to the right side; negative values indicate that responses were located too far to the left side. Therefore, both types of errors (reversal and signed errors) focused on children’s errors on the horizontal dimension, whereas absolute deviations are computed based on children’s *x*- and *y*-coordinates.

### Statistical analyses

Analyses were conducted using IBM SPSS 27. In a first step, we investigated effects of scaling factor and perceptual condition on children’s RTs, by computing repeated measures analyses of variance (ANOVAs) with scaling factor as within-participant variable (5), and sex (2), age group (3), and perceptual condition (3) as between-participants variables. Greenhouse–Geisser corrections for repeated measures analyses were used to account for violations of the sphericity assumption whenever necessary. An alpha level of *p* < 0.05 was considered as significant. With respect to learning RTs, it was expected that children would need longer to explore the maps in the haptic as opposed to the visual conditions (Szubielska & Möhring, 2019). Furthermore, learning RTs may vary as a function of different map size, as larger maps may need more encoding time as opposed to smaller maps (Szubielska et al., 2022a). With respect to children’s scaling RTs, we expected an increase in scaling RTs with higher scaling factor, which would result in a quadratic function (i.e. a V-shaped pattern; e.g. Möhring et al., 2014). If scaling down and scaling up are processed similarly, this V-shaped pattern should show symmetric increases for each scaling direction.

In a second step, we focused on children’s errors in locating the disc. To this end, we examined the frequency of children’s propensity to mix up the left and right side of the space (reversal errors). In accordance with previous spatial scaling research, we corrected for these reversal errors by folding children’s answers in the middle (Huttenlocher et al., 1994; Möhring et al., 2014; Plumert et al., 2019); however, we computed analyses with unfolded *and* folded data to see whether results would differ. Furthermore, we investigated effects of scaling factor and perceptual condition on children’s absolute deviations by computing an analogous ANOVA as above. We expected an increase in absolute deviations with higher scaling factor, which would result in a quadratic function (i.e. a V-shaped pattern; e.g. Möhring et al., 2014), and in particular checked for symmetric increases in each scaling direction. Finally, we examined children’s response strategies when locating the disc using their signed errors. Based on previous research in the visual domain (Huttenlocher et al., 1991, 1994), we expected that children might separate the space into two halves and gravitate towards the midpoint in each half.

## Results

### Children’s learning and scaling RTs

#### Learning RTs

**Fig. 2:**
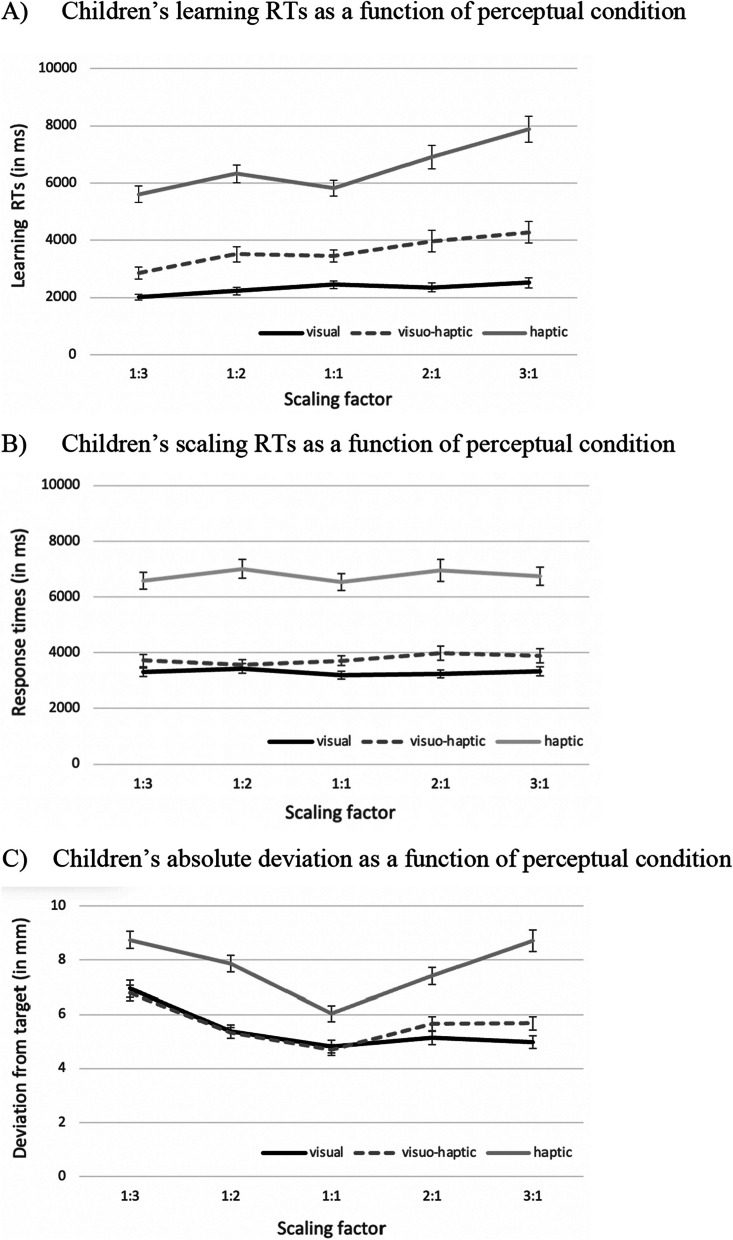
Children’s learning RTs in ms (**A**), their scaling RTs in ms (**B**), and their absolute deviations in mm (**C**) for various scaling factors as a function of different perceptual conditions. Error bars represent standard errors

An outlier analysis for learning RTs revealed that 2.19% of all responses exceeded the criteria of mean ± 3 *SD*s and being below 120 ms (Ishihara et al., 2006; Möhring et al., 2019). These values were excluded and data were collapsed across various locations, but separately for each scaling factor. The ANOVA yielded a significant main effect of perceptual condition (for descriptive and inferential statistics, see Table 3), because children showed higher learning RTs in the haptic condition as compared to the visual conditions (both *p*s < 0.001). In addition, learning RTs of the visuo-haptic condition were higher as compared to the visual condition (*p* < 0.01). The same ANOVA also revealed an effect of age group, which was qualified by a significant interaction between age group and perceptual condition. It was found that 8-year-olds showed higher learning RTs in the haptic condition as compared to the other age groups (both *p*s < 0.01), whereas these younger age groups did not differ (*p* = 0.90). By contrast, learning RTs in the visual conditions did not differ among the age groups (all *p*s > 0.17). The ANOVA also revealed a significant effect of scaling factor, which was best explained by a linear trend, *F*(1, 183) = 84.29, *p* < 0.001, *η*_*p*_^2^ = 0.32. This main effect was qualified by a significant interaction between scaling factor and perceptual condition. As can be seen in Fig. 2A, children’s learning RTs increased linearly with larger size of the map, suggesting that children needed longer to learn about larger maps as compared to smaller maps. This increase seems especially prominent in the haptic condition and less steep in the visual conditions. This impression was underlined by separate ANOVAs for each perceptual condition. There was a significant effect of scaling factor for each perceptual condition which was best explained by linear functions, visual: *F*(1, 64) = 19.80, *p* < 0.001, *η*_*p*_^2^ = 0.24; visuo-haptic: *F*(1, 60) = 30.49, *p* < 0.001, *η*_*p*_^2^ = 0.34, haptic: *F*(1, 59) = 37.28, *p* < 0.001, *η*_*p*_^2^ = 0.39.

**Table 3:** Descriptive and inferential statistics of children’s learning RTs, scaling RTs, and absolute deviations in the spatial scaling task

	Learning RTs (in ms)				Scaling RTs (in ms)				Absolute deviations (in mm)			
	*M* (*SE*)	*F*	*p*	*η*_*p*_^2^	*M* (*SE*)	*F*	*p*	*η*_*p*_^2^	*M* (*SE*)	*F*	*p*	*η*_*p*_^2^
Scaling factor (SF)		**37.75**	** < .001**	**.17**		1.53	.19	.01		**32.34**	** < .001**	**.15**
1:3	3456 (164)				4502 (167)				7.48 (0.19)			
1:2	3973 (188)				4636 (182)				6.17 (0.17)			
1:1	3872 (156)				4445 (159)				5.17 (0.15)			
2:1	4358 (229)				4686 (193)				6.05 (0.17)			
3:1	4833 (252)				4620 (182)				6.42 (0.21)			
Perceptual condition		**82.64**	** < .001**	**.48**		**76.65**	** < .001**	**.46**		**57.08**	** < .001**	**.38**
Visual	2314 (126)				3302 (134)				5.45 (0.17)			
visuo-haptic	3618 (269)				3773 (196)				5.63 (0.14)			
Haptic	6508 (309)				6769 (301)				7.76 (0.18)			
Age group		**5.38**	** < .01**	**.06**		**4.78**	** < .01**	**.05**		2.50	.085	.03
6 years	3821 (350)				4155 (299)				6.46 (0.23)			
7 years	3820 (244)				4475 (230)				6.34 (0.19)			
8 years	4752 (394)				5134 (341)				5.94 (0.20)			
Sex		1.51	.22	.01		0.26	.61	.001		**5.70**	** < .05**	**.03**
Male	4013 (251)				4703 (238)				6.43 (0.17)			
Female	4206 (280)				4420 (224)				6.03 (0.17)			
SF × perceptual condition		**8.30**	** < .001**	**.08**		1.15	.33	.01		**3.63**	** < .001**	**.04**
SF × age group		1.06	.39	.01		0.30	.97	.003		0.95	.47	.01
SF × sex		0.37	.83	.002		0.21	.93	.001		0.98	.42	.01
Perceptual condition × age group		**3.11**	** < .05**	**.06**		**2.86**	** < .05**	**.06**		0.26	.90	.01
Perceptual condition × sex		0.30	.74	.003		0.11	.90	.001		0.25	.78	.003
Age group × sex		0.99	.37	.01		0.54	.58	.01		1.10	.33	.01
SF × perceptual condition × age group		1.52	.09	.03		0.88	.59	.02		0.60	.88	.01
SF × perceptual condition × sex		0.33	.95	.004		0.45	.89	.01		0.29	.97	.003
SF × age group × sex		0.72	.68	.01		1.06	.39	.01		1.76	.09	.02
Perceptual condition × age group × sex		0.27	.89	.01		0.10	.98	.002		0.33	.86	.01
SF × perceptual condition × age group × sex		0.46	.97	.01		0.66	.84	.01		0.93	.54	.02

Standard errors (*SE*) are presented in parentheses. Significant effects are highlighted in bold

*SF* scaling factor

#### Scaling RTs

An outlier analysis for scaling RTs revealed that 2.23% of all responses in the scaling RTs exceeded the criteria of mean ± 3 *SD*s and being below 120 ms^1^ (Ishihara et al., 2006; Möhring et al., 2019). These values were excluded and data were collapsed across various locations, but separately for each scaling factor. The ANOVA yielded a significant main effect of perceptual condition (for descriptive and inferential statistics, see Table 3), because children showed higher scaling RTs in the haptic condition as compared to the visual conditions (both *p*s < 0.001), whereas the visual conditions did not differ (*p* = 0.51). The same ANOVA revealed also an effect of age group, which was qualified by a significant interaction between age group and perceptual condition. Eight-year-olds showed higher scaling RTs in the haptic condition as compared to the other age groups (both *p*s < 0.01), whereas younger age groups did not differ (*p* = 1.0). By contrast, scaling RTs in the visual conditions did not differ among the age groups (all *p*s < 0.20). Given that there were no effects or interactions of scaling factor, it seems that children’s scaling RTs were unaffected by scaling factors and directions (see Fig. 2B).

### Children’s errors and absolute deviations

#### Reversal errors

An inspection of children’s x-coordinates revealed that children produced some reversal errors and located the disc on the other side of the referent space. Not surprisingly, this happened more frequently for the 6-year-olds (13.56%) than for the 7- and 8-year-olds (7.85% and 6.93%, respectively). Furthermore, this occurred more often in the haptic condition (13.48%) as opposed to the visual condition (9.14%) or the visuo-haptic condition (9.41%).^2^ Naturally, these reversal errors produced large variability in children’s absolute deviations. Thus, in accordance with previous research (Huttenlocher et al., 1994; Möhring et al., 2014; Plumert et al., 2019), we gave children credit for their answers and folded children’s answers in the middle, thus accounting for these reversal errors. However, it needs to be noted that analyses with unfolded data revealed similar effects with respect to the variables of interest (i.e. scaling factor, perceptual condition) on children’s absolute deviations.

#### Absolute deviation

An outlier analysis for absolute deviations revealed that 1.74% of all responses exceeded the criteria of mean ± 3 *SD*s. These values were excluded and data were collapsed across various locations, but separately for each scaling factor. The ANOVA yielded a significant main effect of perceptual condition (for descriptive and inferential statistics, see Table 3), because children produced higher absolute deviations in the haptic condition as opposed to both visual conditions (both *p*s < 0.001), with no significant differences between the visual conditions *(p* = 0.95). The same ANOVA revealed also a sex effect, with girls producing lower absolute deviations as compared to boys. The main effect of age group revealed a trend (*p* = 0.085), suggesting that older age groups tended to respond with higher precision. There was also a significant effect of scaling factor, which was best described by a quadratic function, *F*(1, 183) = 69.63, *p* < 0.001, *η*_*p*_^2^ = 0.28. This main effect of scaling factor was qualified by a significant interaction between scaling factor and perceptual condition. As can be seen in Fig. 2C, results indicated a symmetric, quadratic pattern in the haptic condition. By contrast, similar but less symmetric patterns emerged for the visuo-haptic and visual condition. This impression was confirmed by separate ANOVAs for each perceptual condition. For the haptic and visuo-haptic condition, analyses yielded significant effects of scaling factor which were best explained by quadratic functions, haptic: *F*(1, 59) = 30.85, *p* < 0.001, *η*_*p*_^2^ = 0.34, visuo-haptic: *F*(1, 60) = 18.08, *p* < 0.001, *η*_*p*_^2^ = 0.23. On the contrary, the significant effect of scaling factor in the visual condition was best explained by a linear function, *F*(1, 64) = 37.20, *p* < 0.001, *η*_*p*_^2^ = 0.37.

As can be seen in Fig. 2C, in the haptic condition, the increase in children’s absolute deviations was symmetrical for scaling up and scaling down, as confirmed by post hoc comparisons between the symmetric scaling factors (1:3 vs. 3:1 and 1:2 vs. 2:1), revealing no significant differences (both *p*s > 0.92). In the visual and visuo-haptic conditions, children showed an expected increase with higher scaling factors in the scaling up conditions (1:3, 1:2). However, an expected increase in the scaling down condition (2:1, 3:1) was less steep in the visuo-haptic condition and seems non-existent in the visual condition. Post hoc comparisons between the symmetric scaling factors (1:3 vs. 3:1 and 1:2 vs. 2:1) revealed significant differences between the scaling factors 1:3 and 3:1 (both *p*s < 0.05) for both visual conditions, but no differences between the scaling factors 1:2 and 2:1 (both *p*s > 0.990). For both visual conditions, it seems that children produced lower absolute deviations for the largest scaling factor 3:1 (scaling down) as compared to the analogous scaling factor 1:3 (scaling up).

#### Signed errors

**Fig. 3:**
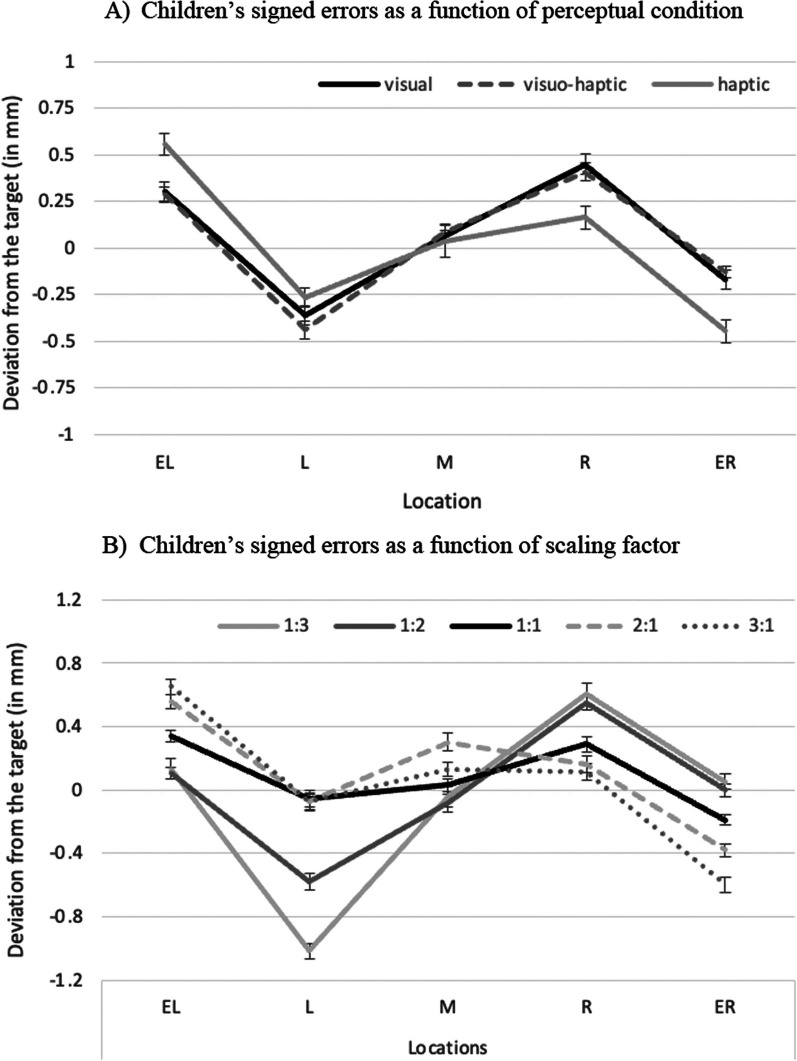
Children’s signed errors for various locations as a function of different perceptual conditions (**A**) and as a function of different scaling factors (**B**). EL = extreme left location, L = left location, M = middle location, R = right location, ER = extreme right location. Error bars represent standard errors. Positive values indicate that the response disc was placed too far to the right side; negative values indicate that responses were located too far to the left side

Whereas children’s absolute deviations reveal information about children’s precision in locating the disc, they cannot reveal potential response strategies and biases. Signed errors allow discovering children’s biases to overt and imagined reference points such as borders or midpoints. Given the scope of the present study on investigating effects of scaling factor and perceptual condition, we focused on illustrating children’s signed errors as a function of scaling factor and perceptual condition. As can be seen in Fig. 3A, children responded highly accurately for the middle location (3), whereas their answers gravitated towards an imagined midpoint on each half of the referent space. This response strategy appeared for every perceptual condition even though deviations were larger in the haptic condition. Therefore, it seems that children have mentally split the space into two halves for each perceptual condition and their answers were biased towards the middle of each half. Figure 3B shows that this gravitation pattern seems true for the scaling factors of scaling up (1:3 and 1:2), but looks quite different for scaling down (2:1 and 3:1). For these latter scaling factors, it seems that children responded accurately for the midpoint and close-to-midpoint locations. For the extreme left and right locations, their responses gravitated towards an imagined midpoint of the entire space.

## Discussion

The current study investigated effects of scaling direction and perceptual modality on children’s spatial scaling. Using a recently developed methodology (Szubielska et al., 2022a), it was found that children learned about target locations in the maps relatively quickly, but needed longer learning times for larger map sizes (Szubielska et al., 2022a). This result was especially pronounced in the haptic condition and likely reflects the sequential encoding process in haptic learning. Additionally, there was a main effect of perceptual condition given that children needed longer to haptically explore the maps as opposed to the visual conditions. In a similar vein, children’s scaling RTs differed between perceptual conditions, with children in the haptic condition showing higher scaling RTs. A closer look at children’s absolute deviations revealed that children of all age groups produced more errors in the haptic as opposed to the visual conditions. Combining these results about RTs and absolute deviations, it seems that children had more difficulties with spatial scaling in the haptic as opposed to the visual modality, which is in line with several adult studies (Ottink et al., 2021; Szubielska et al., 2022a, b).

Additionally, the present design allowed investigating whether children’s RTs and absolute deviations varied as a function of scaling factors. This variation is typically interpreted as an indicator for mental imagery in spatial scaling (cf. Möhring et al., 2014, 2016). Seeing mental imagery in the visual and haptic modality would support functional equivalence of vision and touch and speak for amodal mental representations (Giudice et al., 2011; Loomis et al., 2013). Unfortunately, children’s scaling RTs showed no variation as a function of scaling factors, which remained the same across the three perceptual conditions. This non-significant result was unexpected in the light of previous studies showing increasing RTs with higher scaling factors (at least for scaling up in the visual domain, Möhring et al., 2014, 2018) and makes it hard to pinpoint whether mental imagery was used in our visual and haptic scaling task. In contrast, children’s absolute deviations varied as a function of scaling factors, and at least for scaling up, showed similarity across the three perceptual modalities. Moreover, a look at children’s signed errors indicated similar encoding strategies in each perceptual condition, suggesting that children used comparable response strategies in each perceptual condition. Similar to findings in the visual domain (Huttenlocher et al., 1991, 1994), children mentally divided the space into two categories and gravitated their answers towards an imagined midpoint in each half.

Summing up these findings, it seems that the functional equivalence between vision and touch seems to emerge slowly across development for spatial scaling (Giudice et al., 2011; Loomis et al., 2013). Our findings suggest an analogy of children’s biases when locating the targets across different perceptual modalities which supports the emergence of a functional equivalence. Further support comes from our result showing that children’s absolute deviations varied as a function of scaling factors in different perceptual conditions (particularly for scaling up). Yet, the overall higher RTs and absolute deviations in the haptic condition indicate more difficulties in the haptic domain.

### Scaling up = scaling down?

In addition to exploring effects of perceptual condition, the current study also aimed at investigating whether children’s absolute deviations and scaling RTs were similarly affected by scaling up vs. scaling down. Again, unfortunately, scaling RTs were inconclusive with respect to this question. In contrast, absolute deviations were affected by scaling factors and showed the expected quadratic pattern. That is, absolute deviations increased with higher scaling factors. Interestingly, the symmetry of this quadratic pattern differed between perceptual conditions. In the haptic condition, children’s absolute deviations did not differ between the largest scaling factors in each scaling direction (1:3, 3:1), resulting in a highly symmetric, quadratic function. Thus, it seems that in the haptic condition, children’s absolute deviations increased with higher scaling factors for scaling up *and* scaling down. In contrast, in the visual conditions, children responded more accurately for the scaling factor 3:1 (scaling down) as opposed to scaling factor 1:3 (scaling up). Therefore, for the visual conditions, it seems that children’s absolute deviations increased with higher scaling factors in the scaling up condition whereas absolute deviations remained relatively constant on a low level in the scaling down condition. These results corroborate previous studies in the visual domain showing that children’s errors increased with higher scaling factors when scaling up (e.g. Möhring et al., 2014, 2018). At the same time, our findings qualify these findings and inform us about differences between perceptual modalities and scaling directions.

Children’s signed errors may help to shed light on these results. Children used different response strategies depending on scaling direction when coding locations. When maps were small in the scaling factor condition 1:3 and differences between locations were subtle, children tended to split the entire space into two halves and used midpoints of each half as additional imagined anchor points (cf. Huttenlocher et al., 1994). These mental subdivisions have been shown to improve precision in human’s estimations and are typically used when a fine-grained coding of locations seems more difficult (cf. category adjustment model, Huttenlocher et al., 1991). On the contrary, differences between varying locations could be encoded more easily and quickly in maps with a scaling factor of 3:1. In this respective condition, children tended to consider the map as one spatial category and only absolute deviations for extreme locations were found to gravitate to the midpoint of the entire space. Overall, it seems that children used mental subdivisions of the space, but these mental categories depended on the visibility of the presented map. When visibility did not play a role in the haptic condition, children’s absolute deviations were equally affected by scaling up vs. scaling down, suggesting rather similar underlying processes for each scaling direction. This result is in line with previous studies indicating that a scale translation is compromised when children and adults were not able to see both edges of the space simultaneously (Hund et al., 2020; Plumert et al., 2019). Our findings qualify this conclusion in that visibility seems to affect the scaling process also when spaces are rather small.

### A developmental perspective onto spatial scaling

Similar to several previous studies (Frick & Newcombe, 2012; Gilligan et al., 2018; Möhring et al., 2018; Plumert et al., 2019), our results showed a trend to respond with increased precision when localizing the targets with age. This was reflected by a higher propensity of 6-year-olds to mix up the left and right side of the space, but similarly by higher absolute deviations in younger age groups. By contrast, our findings indicated that learning and scaling RTs were higher in the 8-year-olds as opposed to the 6-year-olds. At first sight, this result seems unexpected given the improved information processing speed across development (for a meta-analysis, see Kail, 1991). This higher precision along with higher RTs may suggest a speed-accuracy trade off in 8-year-olds. However, our findings indicated that this age difference results predominantly from children’s responses in the haptic perceptual condition. It seems that 8-year-olds have spent particularly more time exploring the map and scaling spatial information in the haptic condition as opposed to younger age groups. This finding might be explained by 8-year-olds being more aware that the haptic condition is a rather unusual condition and thus requires more cognitive resources and allocation of study time (Dufresne & Kobasigawa, 1988, 1989). Consequently, age-related differences in these RTs may be reflective of different meta-cognitive strategies between the age groups.

### Strengths and limitations

We consider it a strength that our design enabled to assess scaling direction using different scaling factors for each direction in a within-participant design. Moreover, our sample size was adequately powered and larger as compared to previous spatial scaling studies (Frick & Newcombe, 2012; Hund et al., 2020; Möhring et al., 2014, 2015, 2018; Plumert et al., 2019). Furthermore, there was only minimal dropout even though one-third of the children was asked to wear a blindfold during the test trials, demonstrating that the task was entertaining and feasible for the present age range. Finally, the present study paved the way for assessing spatial scaling not only in the visual domain but allowed comparing scaling across different perceptual domains, shedding light into similarities and differences across modalities.

In addition to these strengths, several limitations warrant mention. We consider it a limitation that the scaling RTs were inconclusive with respect to effects of scaling factors and scaling directions. Even though we used a two-stage methodology and took care to separate learning about the map from scaling and localizing the disc on the referent space (for a discussion about the necessity, see Szubielska & Möhring, 2019), a look at children’s scaling RTs showed large variability in the data. Future studies may use video recordings of children’s responses and code children’s scaling RTs after each testing session. Furthermore, the present study used only one-dimensional target distributions and future studies may use two-dimensional distributions or maybe even three-dimensional spaces to investigate whether our results can be replicated in more complex, yet highly realistic scenarios. Moreover, in the current study, we were restricted to relatively small spaces that can be explored haptically in a reasonable amount of time while still allowing to investigate different scaling directions in a single comprehensive design. Future studies may use larger spaces while still being able to assess different scaling directions and perceptual domains. Another limitation concerns our sample with a selected age range between 6 and 8 years, with many children coming from families with high educational attainment. It remains unclear whether findings can be generalized to even younger and older age groups and to samples with lower educational attainment. Our analyses revealed a sex difference, with girls responding more accurately as opposed to boys. This result was unexpected in the light of the well-known male advantage in spatial skills, particularly in tasks involving mental rotation (for a review, see Levine et al., 2016) and non-significant differences between girls and boys in previous spatial scaling tasks (6-year-olds in Frick & Newcombe, 2012; 6- and 7-year-olds in Möhring et al., 2018; but see Gilligan et al., 2018). Future studies may further explore this sex difference in children’s spatial scaling. Finally, it was not feasible to examine perceptual condition as within-participant variable considering the number of trials in the experimental session and the age of our young participants.

## Conclusions

The present study investigated spatial scaling in children aged 6–8 years and assessed effects of scaling direction and perceptual condition on children’s performance. Our findings indicated that children showed similar response biases in the haptic and visual perceptual conditions. Similarly, children produced higher absolute deviations when they needed to scale spatial information to a larger extent (with stronger effects found for scaling up). Whereas this result points to the usage of mental representations in visual and haptic scaling, children’s scaling RTs were inconclusive, precluding conclusions about the usage of mental imagery in different perceptual domains. Given that children produced higher errors and RTs in the haptic condition, it seems that the functional equivalence between vision and touch seems to emerge slowly across development for spatial scaling (Giudice et al., 2011; Loomis et al., 2013).

With respect to scaling direction, our results suggested no variations in children’s scaling RTs but in their absolute deviations. In the visual conditions, it was found that scaling down resulted in lower absolute deviations as opposed to scaling up which is in line with previous studies (Hund et al., 2020; Plumert et al., 2019; Siegel et al., 1979), even though we took care to keep the referent space constant and small-sized in both scaling directions. Overall, it seems that the visibility of maps affected children’s spatial scaling which seems to hold for rather small map sizes but also for very large spaces in which both edges of the space cannot be encoded simultaneously (Hund et al., 2020; Plumert et al., 2019). When visibility did not factor into the scaling process as in the haptic condition, it was found that scaling processes were identical for each scaling direction. Thus, our findings shed light onto potential influential variables such as the size of spatial layouts on children’s spatial scaling, which might be applied to practical problems such as creating suitable material in educational and professional contexts. Building upon these findings, it seems that scale translations in the visual domain are easily interfered when visibility of the maps is constrained. Future studies may further probe effects of different absolute sizes on spatial scaling and may even extend the scope of scaling studies to spaces that go beyond our perceptual skills and challenge our imagery skills (e.g. scaling down information to nanoscale, scaling up information to the solar system).

## Data Availability

Data of the present study will be made publicly available via the Open Science Framework (OSF) and can be accessed at https://osf.io/w6m82/.

## Footnotes

1 For scaling RTs, it never happened that RTs were lower than 120 ms; for learning RTs, this occurred for 4 cases out of 5025 trials (< 0.01%).

2 We checked whether handedness was related to children’s propensity to show reversal errors and found that this was not the case, *r*(191) = .023, *p* = .752.
